# Characterization of γ-H2AX foci formation under alpha particle and X-ray exposures for dose estimation

**DOI:** 10.1038/s41598-022-07653-y

**Published:** 2022-03-08

**Authors:** Ui-Seob Lee, Dong-Hyun Lee, Eun-Hee Kim

**Affiliations:** Department of Nuclear Engineering, Seoul National University, 1 Gwanak-ro, Gwanak-gu, Seoul, 08826 Republic of Korea

**Keywords:** Computational models, Computational biophysics

## Abstract

DNA double-strand break (DSB) induction is one of the phenotypes of cellular damage from radiation exposure and is commonly quantified by γ-H2AX assay with the number of excess fluorescent foci per cell as the main component. However, the number of foci alone may not fully characterize the state of DNA damage following exposures to different radiation qualities. This study investigated the feasibility of utilizing the focus size distribution and dephosphorylation rate of γ-H2AX to identify the type of causative radiation and dose. Human lung epithelial cells and mouse vascular endothelial cells were used to observe the expression changes of γ-H2AX foci due to alpha particle and X-ray exposures. Results showed that the average number of excess foci per cell linearly increased with the dose. The focus size distribution showed a consistent pattern depending on the causative radiation type. Three criteria for the identification of causative radiation type were derived from experimental focus size distributions and were validated in blind testing with correct identification of 27 out of 32 samples. The dose could be estimated based on the proportionality constant specific to the identified radiation type with a difference of less than 15% from the actual value. The different dephosphorylation rates of γ-H2AX produced from alpha particle and X-ray exposures were effectively utilized to determine the individual dose contributions of alpha particles and X-rays under mixed beam exposure. Individual doses were estimated to have differences of less than ~ 12% from actual values.

## Introduction

Radiation affects cells in various ways, including DNA damage. DNA double-strand break (DSB) is one of the key indicators of biological damage and is mainly detected by γ-H2AX immunofluorescence detection assay. The γ-H2AX signals expressed as bright spots (foci) through fluorescence microscopy indicate DNA DSB production, and each γ-H2AX focus corresponds to a single DNA DSB production site at low linear energy transfer (LET) radiation exposure. The number of those spots (γ-H2AX foci) reflects DNA DSB quantity^1^ and linearly increases with the radiation dose^2,3^.

High-LET radiation is more effective in causing DNA damage than low-LET radiation. However, the number of foci induced by high-LET radiation was not significantly different from that by low-LET radiation for the same dose^4–6^, and more foci were observed from low-LET radiation exposure than from high-LET radiation exposure for the same dose^7,8^. High-LET particles deliver energies at a high density and thus form closely spaced foci^9^ that may overlap and be counted as one focus^10^. The γ-H2AX focus containing overlapping foci is larger than an isolated single focus. Since alpha particles can deliver a high dose even with fewer particles, the fluence is much lower than that of photons at the same dose. Thus, fewer γ-H2AX foci can be observed. In consequence, the superior efficiency of high-LET radiation in inducing DNA damage can be identified with large γ-H2AX foci rather than with a large number of foci.

A γ-H2AX focus formed with clustered DSBs may be distinguished from an isolated single focus in dephosphorylation rate. After DSB repair, the nearby γ-H2AX proteins are dephosphorylated, and the number of γ-H2AX foci decreases^2^. The dephosphorylation rate indicates the DNA repair capacity and inversely corresponds to the radiosensitivity of cells^11,12^. Clustered DSBs are less suitable for repair than a simple DSB^13^. The foci generated by high-LET radiation showed a slower reduction over time compared with those produced by low-LET radiation^4,8,14–16^. Alpha particles of high-LET most probably cause complex or dense DNA damages.

This study aims to investigate the feasibility of judging the causative radiation type and estimating the dose based on the pattern of γ-H2AX foci formation and dephosphorylation. The size distribution and dephosphorylation rate of γ-H2AX foci were chosen as the key parameters.

## Results

### γ-H2AX foci production

**Figure 1:**
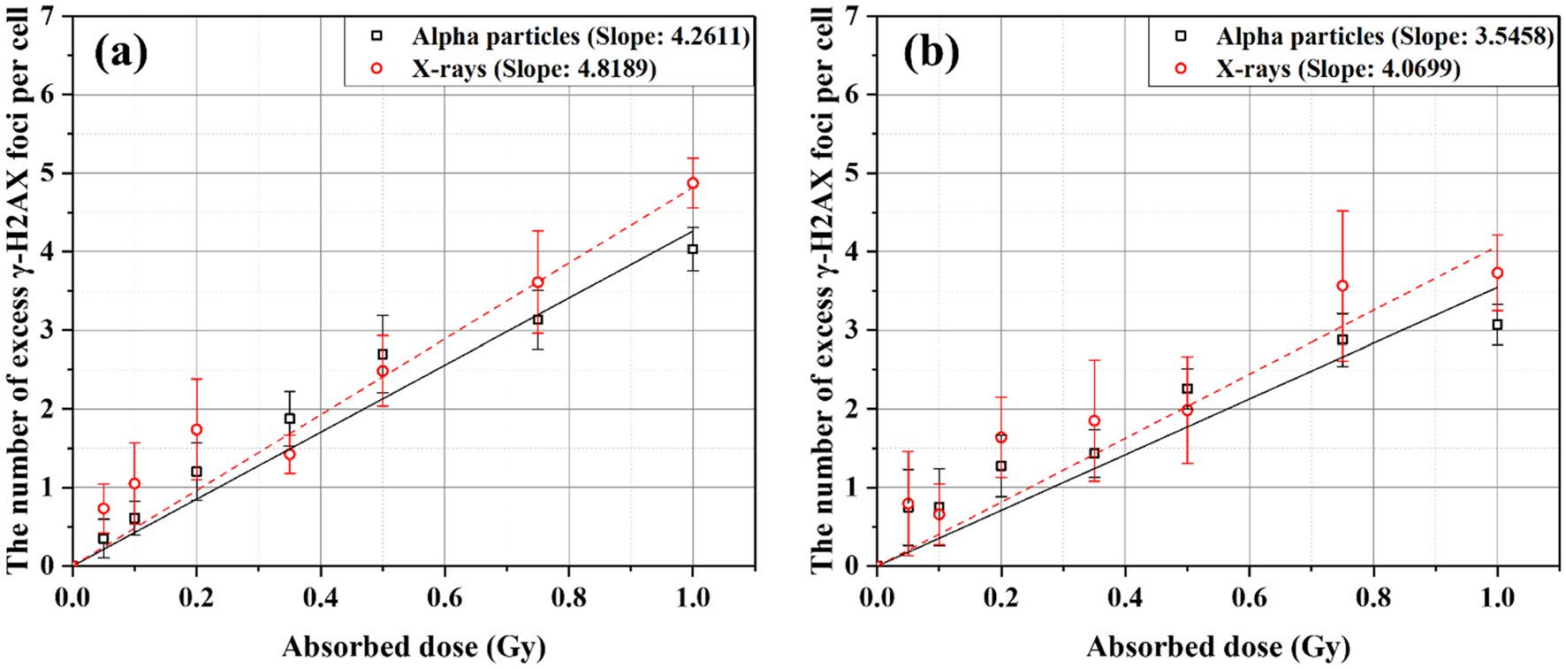
The numbers of excess γ-H2AX foci per cell in (**a**) BEAS-2B cells and (**b**) SVEC4-10EHR1 cells due to exposures to alpha particles (squares) and X-rays (circles) at 1 h post-irradiation. Linear fitting was made for each data set from exposures to alpha particles (solid) and X-rays (dashed) (R^2^ > 0.95). The slopes are given in “the number of excess foci per cell per Gy”. Each data point was obtained from four independent experiments.

Figure 1 presents the numbers of excess foci per cell (FPC) in BEAS-2B and SVEC4-10EHR1 cells irradiated with alpha particles and X-rays at doses of 0.05–1 Gy. The numbers of spontaneous FPC in control BEAS-2B cells were 0.513 and 0.560 at alpha particle and X-ray experimental environments, respectively, and those in control SVEC4-10EHR1 cells were 0.572 and 0.569, respectively. The number of excess FPC measured an hour after irradiation increased with the dose in both cell lines from either alpha particle or X-ray exposure. No substantial difference in the number of excess FPC due to alpha particle and X-ray exposures was found in both cell lines. Data points of the number of excess FPC were fitted to linear functions with R^2^ values over 0.95.

**Figure 2:**
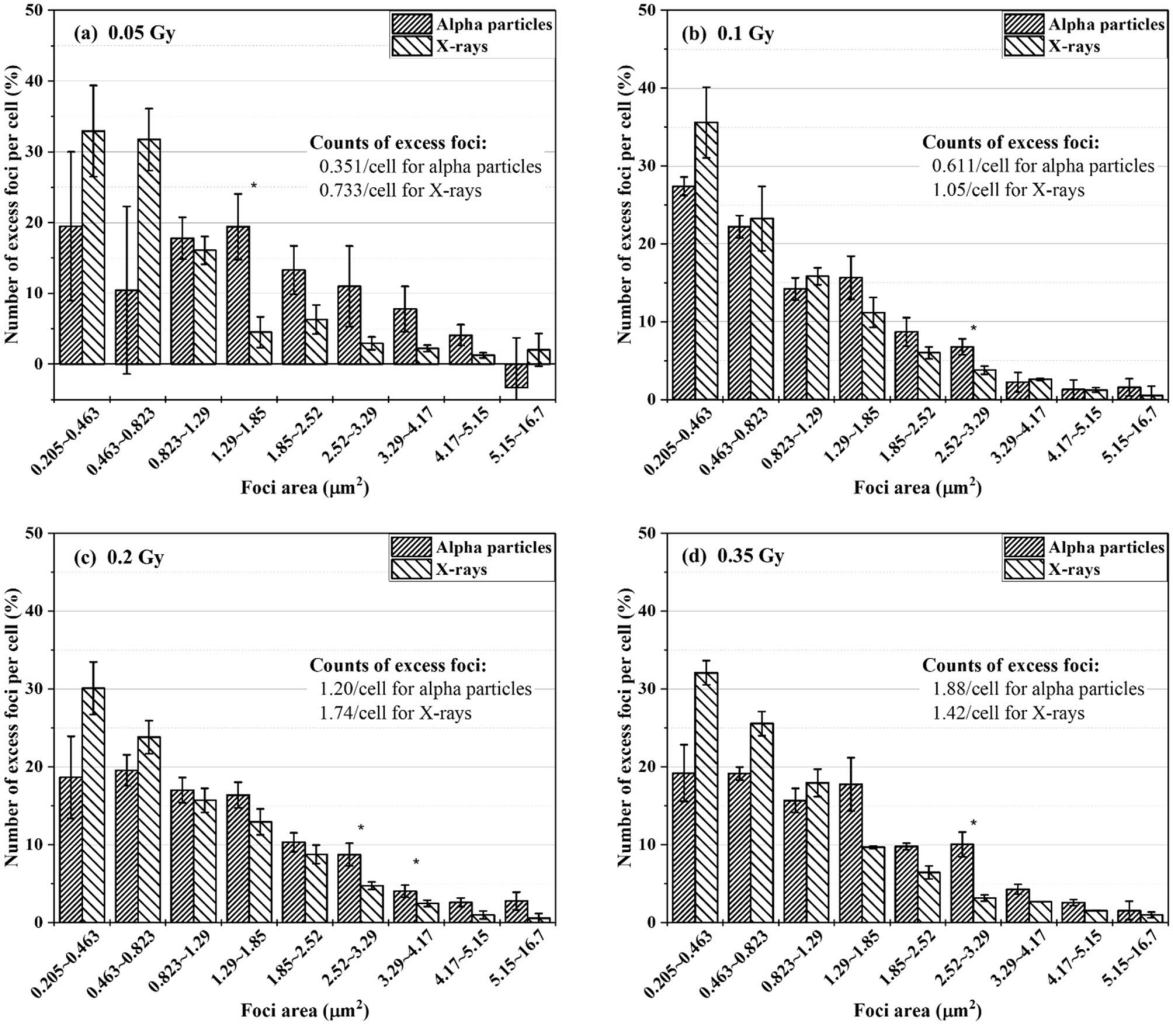

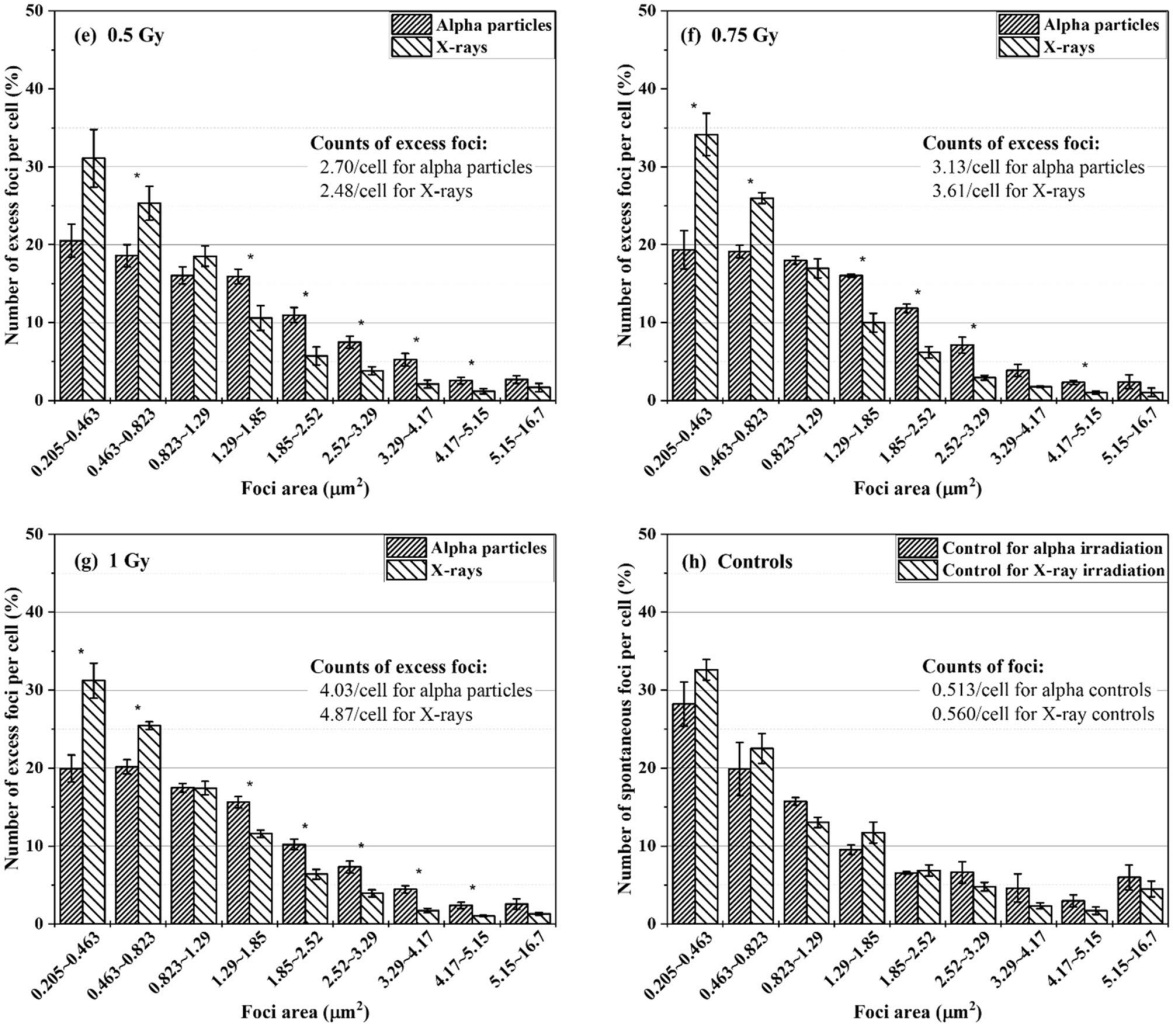

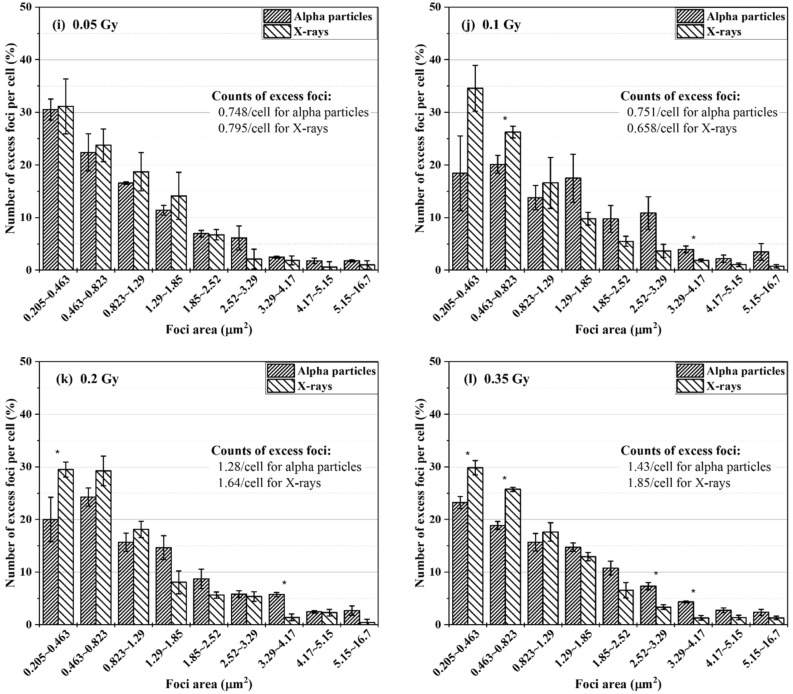

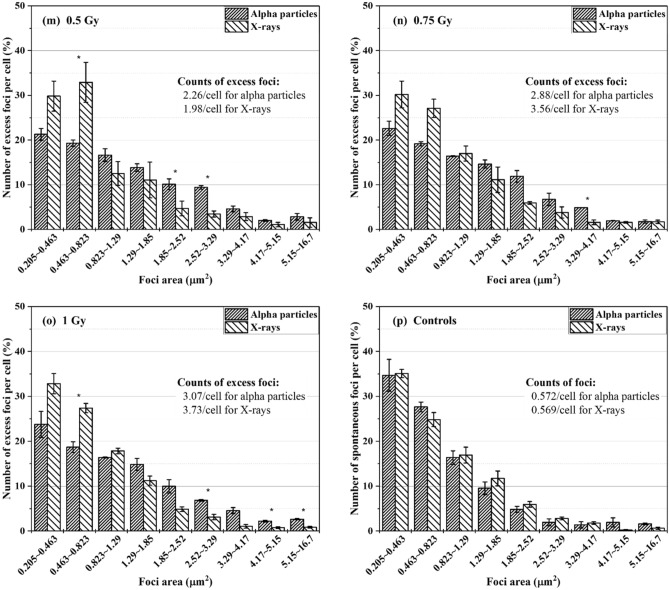
The size distributions of excess γ-H2AX foci in BEAS-2B (**a**–**g**) and SVEC4-10EHR1 (**i**–**o**) cells exposed to 0.05 to 1 Gy of alpha particles and X-rays at 1 h post-irradiation as compared to the size distributions of spontaneous γ-H2AX foci in control BEAS-2B (**h**) and SVEC4-10EHR1 (**p**) cells. The data was collected an hour after irradiation. The asterisks indicate the size groups for which the percentage significantly (p < 0.05) differs depending on the radiation type. Each data point was obtained from four independent experiments.

Foci data collected from alpha particle and X-ray exposures at doses of 0.05–1 Gy were characterized in terms of size distribution. The cells had an hour of repair time. The excess foci were registered for each size group. Figure 2 shows the percentage of the number of excess FPC belonging to individual size groups in BEAS-2B cells (Fig. 2a–g) and SVEC4-10EHR1 cells (Fig. 2i–o). Figure 2h,p present the size distributions of spontaneous foci in control BEAS-2B and SVEC4-10EHR1 cells, respectively. The asterisks indicate the size groups with significantly different (p < 0.05) percentage depending on the radiation type. For BEAS-2B cells, the radiation type-dependency of the size distribution is not substantial at low doses but becomes apparent with improved statistics at high doses (> 0.5 Gy). SVEC4-10EHR1 cells responded with less significant dependency of the size distribution on the radiation type compared with BEAS-2B cells. For both cell lines and at all doses, the size distribution due to alpha particle exposure shifted to large size groups compared with that due to X-ray exposure. Table 1 summarizes the average sizes of foci expressed in both cell lines due to alpha particle and X-ray exposures. At doses between 0.2 and 1 Gy, the mean foci sizes significantly differed (p < 0.05) between alpha particle and X-ray exposures in both cell lines. For each radiation type, the average foci size was consistent regardless of dose.

**Table 1:** The average sizes of γ-H2AX foci expressed in BEAS-2B and SVEC4-10EHR1 cells due to alpha particle and X-ray exposures at doses of 0.05 to 1 Gy.

Cell	Dose (Gy)	Average size of foci (μm^2^)		Difference (%)	p value
		Alpha particles	X-rays		
BEAS-2B	0.05	1.37^(1)^ ± 0.45	1.11^(2)^ ± 0.20	23.4^(3)^	0.62^(4)^
	0.1	1.31 $\pm$ 0.09	1.05 $\pm$ 0.11	24.8	0.12
	0.2	1.64 $\pm$ 0.12	1.13 $\pm$ 0.07	45.1	0.01
	0.35	1.56 $\pm$ 0.04	1.10 $\pm$ 0.06	41.8	0.03
	0.5	1.64 $\pm$ 0.10	1.15 $\pm$ 0.13	42.6	0.03
	0.75	1.57 $\pm$ 0.15	1.04 $\pm$ 0.05	51.0	0.06
	1	1.64 $\pm$ 0.12	1.12 $\pm$ 0.05	46.4	0.01
SVEC4-10EHR1	0.05	1.27 $\pm$ 0.04	1.07 $\pm$ 0.07	18.7	0.01
	0.1	1.73 $\pm$ 0.23	1.02 $\pm$ 0.06	69.6	0.05
	0.2	1.56 $\pm$ 0.06	1.07 $\pm$ 0.04	45.8	< 0.01
	0.35	1.56 $\pm$ 0.04	1.12 $\pm$ 0.02	39.3	< 0.01
	0.5	1.62 $\pm$ 0.09	1.12 $\pm$ 0.16	44.6	0.04
	0.75	1.50 $\pm$ 0.08	1.16 $\pm$ 0.07	29.3	0.03
	1	1.55 $\pm$ 0.09	1.00 $\pm$ 0.07	55.0	0.03

Percent difference (3) = {(1) − (2)}/(2) × 100.

p value (4) from two-tailed Student’s t-test with (1) and (2).

### γ-H2AX dephosphorylation rate

**Figure 3:**
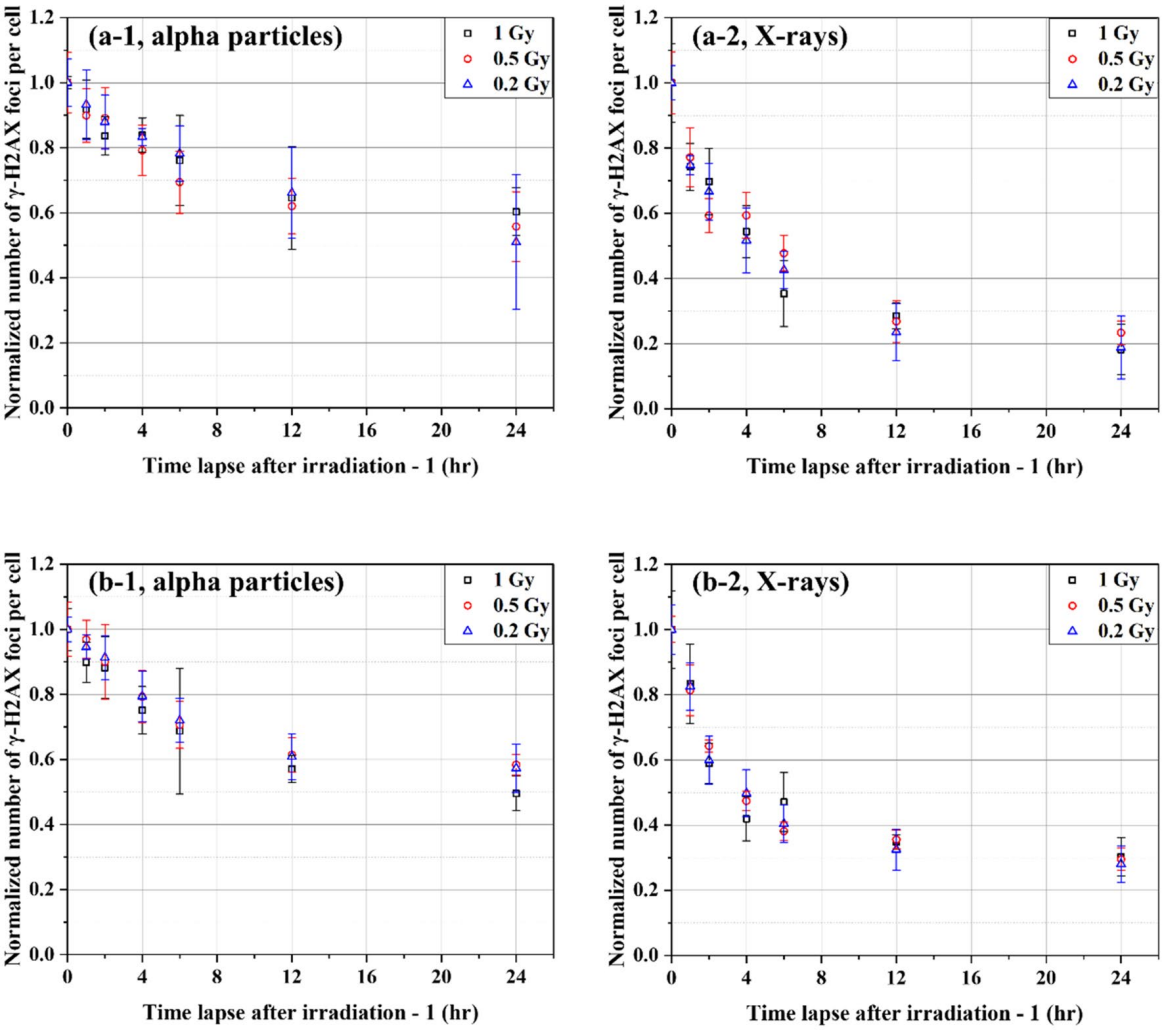
The normalized numbers of excess γ-H2AX foci per cell over time in (**a**) BEAS-2B and (**b**) SVEC4-10EHR1 cells irradiated at 0.2, 0.5, and 1 Gy with alpha particles and X-rays. Each data point was obtained from four independent experiments.

**Figure 4:**
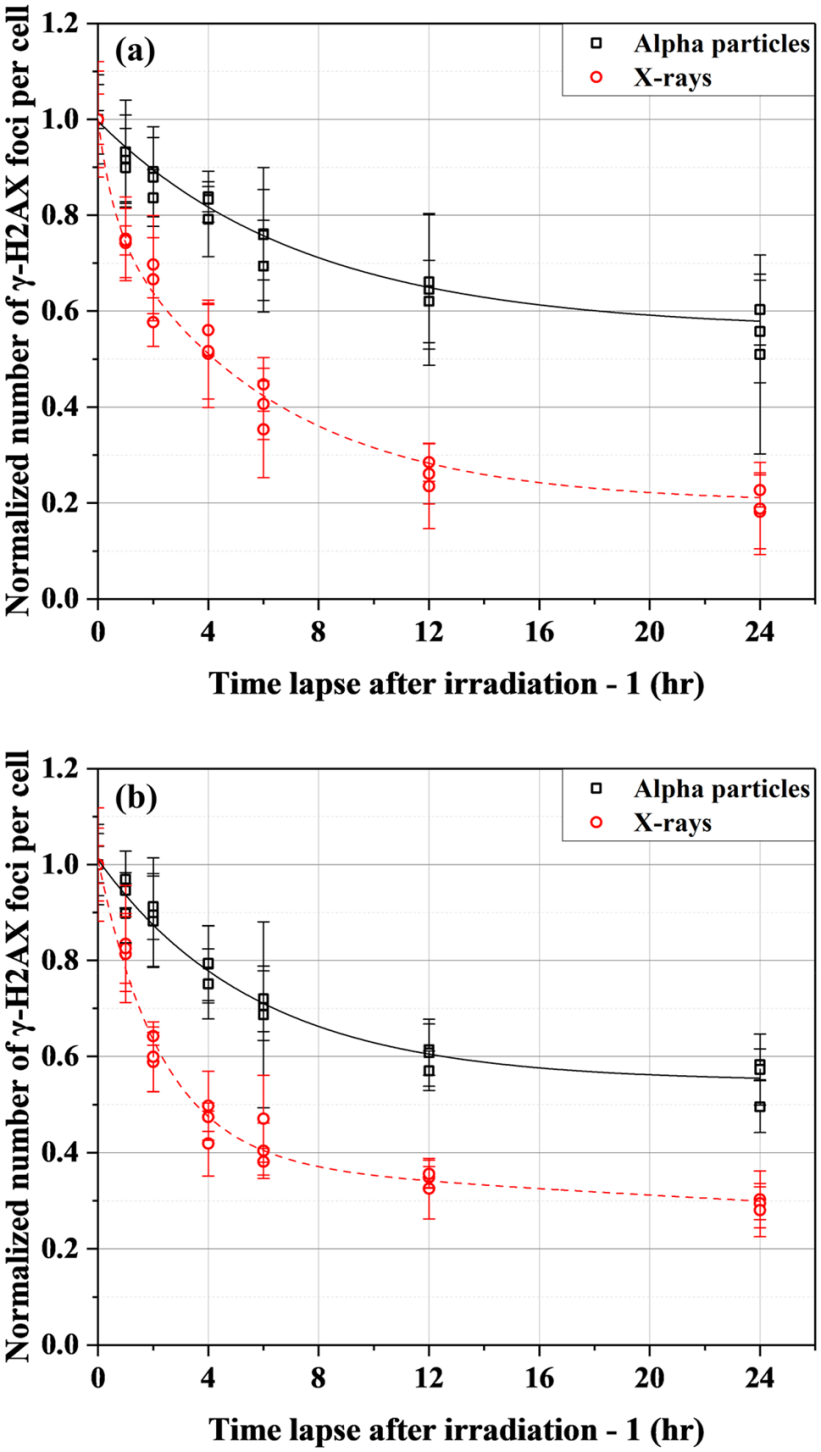
The normalized numbers of excess γ-H2AX foci per cell over time in (**a**) BEAS-2B and (**b**) SVEC4-10EHR1 cells irradiated with alpha particles and X-rays. Each data point was obtained from four independent experiments.

Figure 3 shows the normalized numbers of excess γ-H2AX FPC decreasing over time in BEAS-2B and SVEC4-10EHR1 cells after irradiation at 0.2, 0.5 and 1 Gy with alpha particles and X-rays. Regardless of dose level, the data points for each cell line converged to the same curve specific to the radiation type. Figure 4a,b present the fitting curves specific to individual radiation types for BEAS-2B and SVEC4-10EHR1 cell lines, respectively. Table 2 lists the constants for the fitting functions of a standard form ${\text{A}}_{1}{{\text{e}}}^{-{\text{t}}/{\text{B}}_{1}}\text{+}{\text{A}}_{2}{{\text{e}}}^{-{\text{t}}/{\text{B}}_{2}}\text{+}{\text{A}}_{3}{{\text{e}}}^{-{\text{t}}/{\text{B}}_{3}}$ where *B*_*i*_ is the individual dephosphorylation rate, and *A*_*i*_ indicates the corresponding contribution to the total number of excess γ-H2AX foci. The value of (*ln 2* $\times$ *B*_*i*_) is the half-life for loss of excess γ-H2AX foci with the corresponding dephosphorylation rate. In both cell lines, the loss of excess γ-H2AX foci was observed within a day after irradiation. γ-H2AX dephosphorylation proceeded faster in the cells exposed to X-rays than in those exposed to alpha particles with smaller *B*_*i*_ values (0.557, 6.04 < 7.53 for BEAS-2B and 2.27 < 5.74 for SVEC4-10EHR1).

**Table 2:** The constants for exponentially decaying fitting functions of a standard form ${\text{A}}_{1}{{\text{e}}}^{-{\text{t}}/{\text{B}}_{1}}\text{+}{\text{A}}_{2}{{\text{e}}}^{-{\text{t}}/{\text{B}}_{2}}\text{+}{\text{A}}_{3}{{\text{e}}}^{-{\text{t}}/{\text{B}}_{3}}$.

Cell line	Radiation	Constants*							
		*A*_*1*_	*B*_*1*_	*ln 2* $\times$ *B*_*1*_	*A*_*2*_	*B*_*2*_	*ln 2* $\times$ *B*_*2*_	*A*_*3*_	*B*_*3*_
BEAS-2B	Alpha particles	0.435	7.53	5.22	0.273	3.08 $\times$ 10^4^	2.13 $\times$ 10^4^	0.288	4.12 $\times$ 10^146^
	X-rays	0.197	0.557	0.386	0.603	6.04	4.19	0.200	3.85 $\times$ 10^86^
SVEC4-10EHR1	Alpha particles	0.462	5.74	3.98	–	–		0.548	1.07 $\times$ 10^141^
	X-rays	0.628	2.27	1.57	–	–		0.383	98.1

*B_*i*_’s and (*ln 2 *$\times$* B*_*i*_)’s in hr.

## Discussion

### Number of γ-H2AX foci as a dose index

Figure 1 depicts the linear proportionality of the number of excess γ-H2AX foci to radiation dose of up to 1 Gy. The specificity of proportionality constant (slope of the fitting line) to the radiation type is discernible, but the difference is not significant (p > 0.05). Other studies^4–6^ observed minimal difference in the number of foci upon exposure to X-ray and alpha particles. Given that alpha particles have larger relative biological effectiveness than X-rays^5^, the number of foci alone cannot convey the radiation quality.

### Size distribution of γ-H2AX foci

Biological effectiveness in terms of DNA DSB production cannot be estimated by the amount of foci formation. Focus size could indicate the severity of cellular response with differential data between alpha particle and X-ray exposures. The size of γ-H2AX foci in relation to radiation quality has been widely reported. Leatherbarrow et al.^4^ showed that alpha particles produce 1.5 times larger foci than γ-rays (0.3 μm^2^ due to alpha particles and 0.2 μm^2^ due to X-rays), and Antonelli et al.^8^ stated 66% size difference (2.1 μm^2^ due to alpha particles and 1.26 μm^2^ due to X-rays). Foci generated with lithium and nitrogen ions are twice larger than those with low-LET radiations^7,17^. With regard to the difference in the average size of γ-H2AX foci, our observation is consistent with previous works. The average size of foci due to alpha particle exposure was approximately 40–50% larger than that due to X-ray exposure (Table 1). The present study further shows that the causative radiation is distinct in the size distribution of γ-H2AX foci (Fig. 2). At doses up to 1 Gy, both cell lines expressed more foci in small size groups when exposed to X-rays than alpha particles. Both cells also expressed greater portions of foci in large size groups when exposed to alpha particles than X-rays. For foci expressed at 0.05 Gy, the size distribution pattern was inconsistent. Large foci are formed due to clustered DNA DSBs, which indicate high biological effectiveness^18^.

Large γ-H2AX foci from high-LET radiation exposure are generated due to the overlap of nearby foci or complex DNA damage. In experiments with cells in vitro and high-LET particles delivered perpendicular to the cell layer, the microscope views the cells in parallel to the beam track inside the cells unless the cells are detached from and reattached to the dish bottom^19–21^. In this view direction, the foci aggregated near the straight track of an alpha particle are visualized as a single large focus^4,6–8,17^. When the irradiation direction is not perpendicular to the cell layer, a microscope views the clustered γ-H2AX foci, that encompass multiple, small foci closely located along the track of a charged particle, and the distant simple foci attributed to low-LET delta-rays^22,23^.

In this study, cells were exposed to alpha particles in one direction from the bottom, but were realigned on a slide glass via cyto-centrifugation after exposure. When the microscope viewed the cells on the slide glass, the alpha tracks in the hit cells were oriented in a random direction. Hence, the foci formed along the alpha track could be viewed separately. The average size of foci measured in the present work was consistent with that reported in other studies where cells were visualized without realignment after irradiation. Hence, we conclude that clustered or simple individual γ-H2AX foci formed due to alpha tracks have little overlap in microscopic images taken in the track direction. In addition, the average size of foci was almost unchanged at doses of 0.2–1 Gy (Table 1), implying that the dose is reflected in the total number of foci, not in their size. Figure 1 depicts the dose-dependency of the number of excess foci. Given that individual foci represent individual spots of DNA damage, large foci are most likely due to clustered DNA damage.

The foci size distribution would change due to dephosphorylation of γ-H2AX over time after radiation exposure^8,15^ and hence the quantitative comparison may perform differently depending on the time-point of foci analysis. Since simple DSBs (indicted by small foci) are more repairable than clustered DSBs (indicated by large foci)^8,10^, it is presumed that the size distribution shifts to larger groups over time, similar to the result in Costes et al.^10^.

### Feasibility test of identifying the causative radiation type and dose based on the focus size distribution

Figure 2 depicts a consistent pattern in both cell lines, that is, the γ-H2AX foci shifted to large size due to alpha particle exposure. Table 1 shows that the average size of γ-H2AX foci due to alpha particle exposure was larger than that due to X-ray exposure. The average size of foci in each cell line varied with the causative radiation type but remained approximately the same at doses between 0.2 and 1 Gy. In this section, the feasibility of identifying the causative radiation type based on the cell-specific reference focus size distribution was discussed.

**Figure 5:**
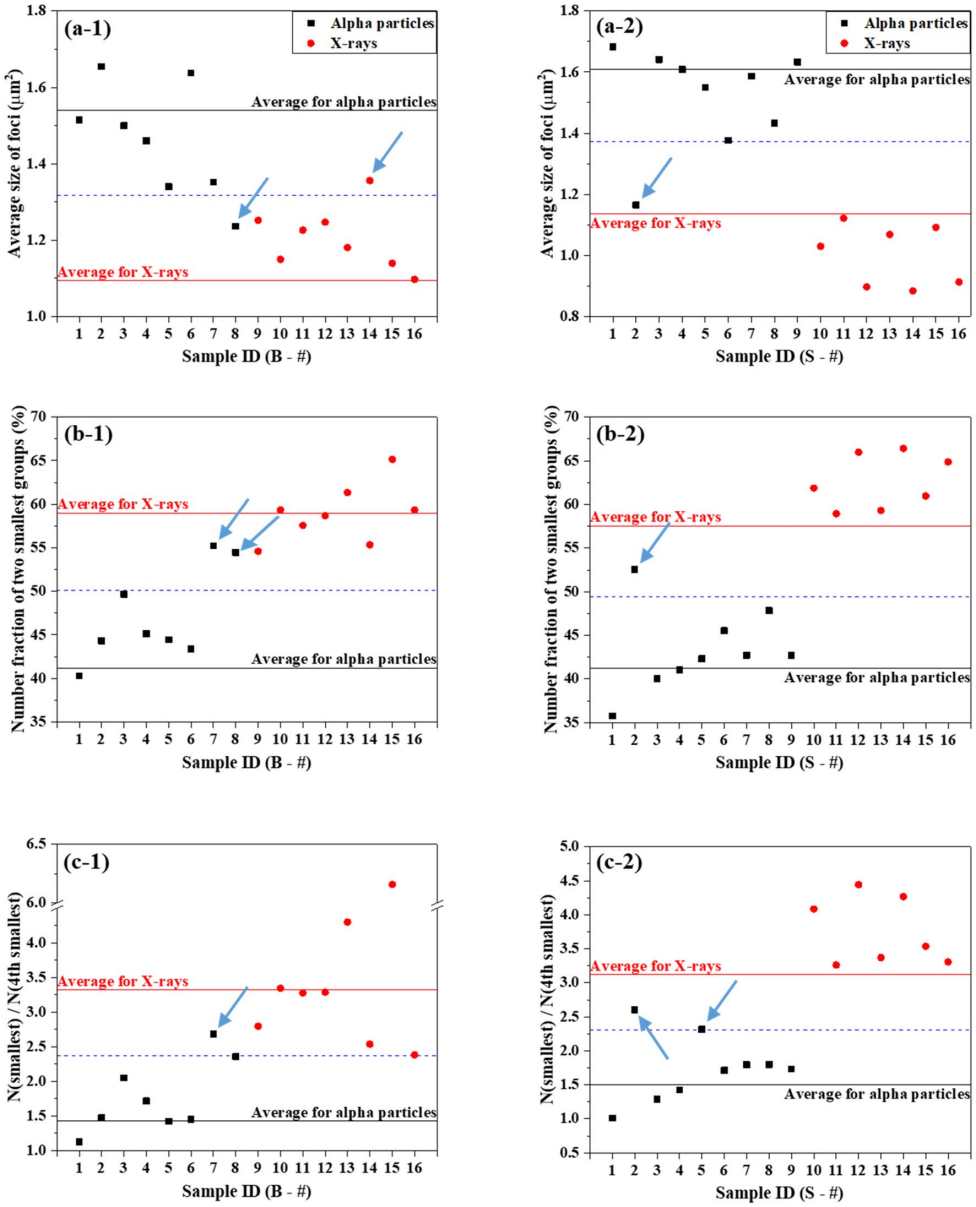
Judgment of the causative radiation type based on γ-H2AX foci size distribution by three criteria: (**a**) the average size of foci, (**b**) the percentage of foci that belong to two smallest groups and (**c**) the ratio of the number of foci per cell in the smallest group to that in the fourth smallest group. Three criterion values from alpha particle (black filled squares) and X-ray (red filled circles) exposures for each of 16 BEAS-2B (**a-1**,**b-1**,**c-1**) and 16 SVEC4-10EHR1 (**a-2**,**b-2**,**c-2**) test samples are displayed in contrast to the reference values (black and red lines for alpha particle and X-ray exposures, respectively). Arrows point to wrong judgments.

The following observation points were chosen as criteria to identify the causative radiation: ① the average size of foci, ② the percentage of foci that belong to the two smallest size groups (0.205–0.823 μm^2^) and ③ the ratio of the number of excess FPC in the smallest group (0.205–0.463 μm^2^) to that in the fourth smallest group (1.29–1.85 μm^2^). The reference value related to each criterion was derived from the cell-specific size distributions upon 0.2–1 Gy exposure as shown in Fig. 2. Black and red lines in Fig. 5 indicate the cell-specific (BEAS-2B in a-1, b-1 and c-1; SVEC4-10EHR1 in a-2, b-2 and c-2) reference values relating to alpha particle and X-ray exposures, respectively. Significant differences between the reference values for alpha particle (black line) and X-ray (red line) exposures were confirmed at 99% confidence level (p values < 0.0007) in all six categories (three criteria per cell line × 2 cell lines) by a two-tailed t-test.

The causative radiation type can be determined as follows: (1) when cells (BEAS-2B or SVEC4-1EHR1) are exposed to unknown radiation, three criterion values of ①, ② and ③ are derived by analysing the size distribution of γ-H2AX foci in the irradiated cells; and (2) the causative radiation is judged by the proximity of a sample criterion value to the corresponding reference value. In the present study, sixteen sample sets of focus size distributions for each of BEAS-2B and SVEC4-10EHR1 cells were prepared to demonstrate the scheme. Black and red dots in Fig. 5 are the criterion values derived from test samples. The arrows indicate the test samples that were judged wrong in blind testing. Correct judgment of the causative radiation type was made by all three criteria, for 81% (13 out of 16 cases) of test sets with BEAS-2B cells and 87% (14 out of 16 cases) of test sets with SVEC4-10EHR1 cells.

**Figure 6:**
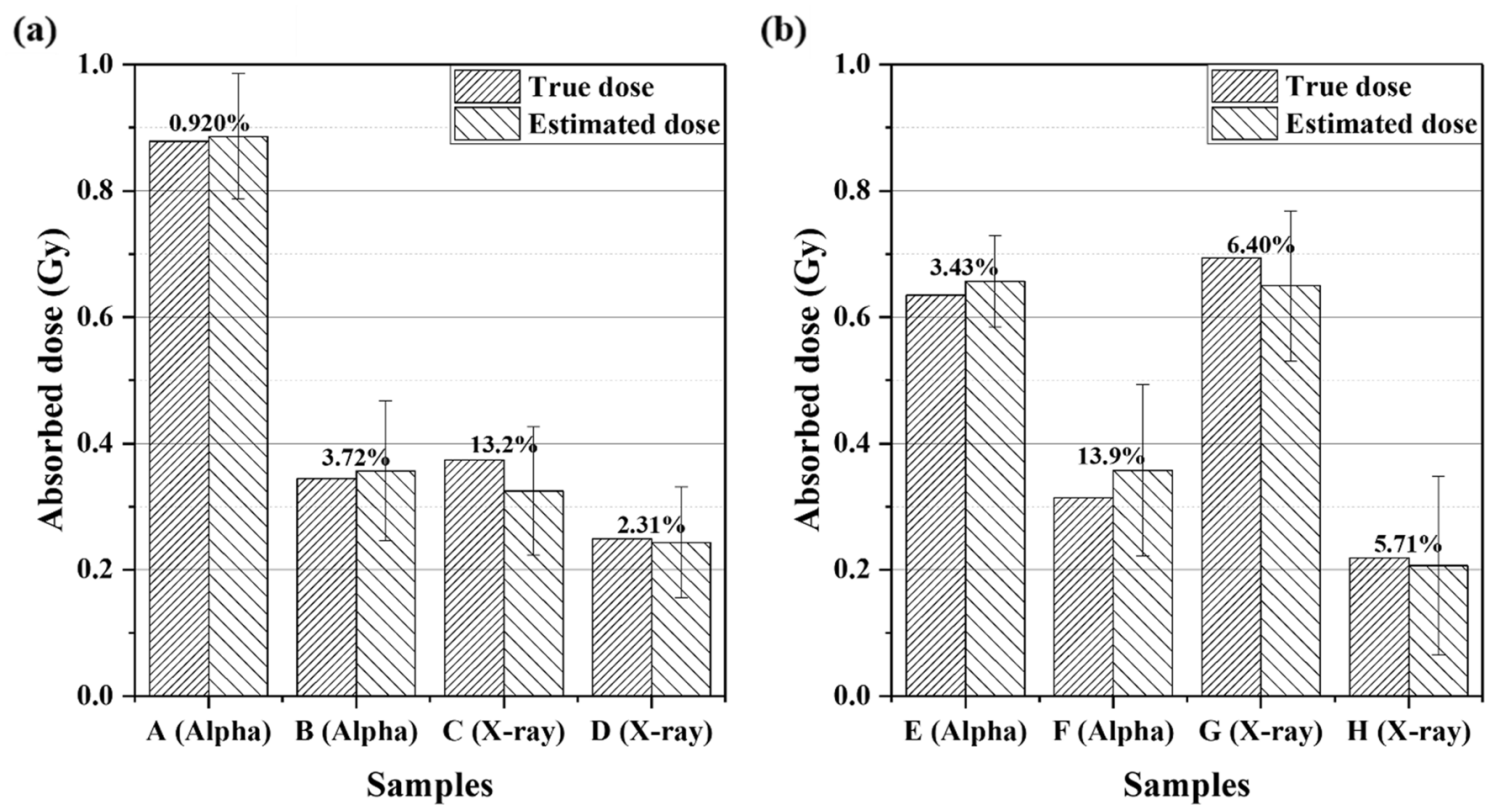
Mean dose estimates for test samples according to the radiation type-specific linear fitting functions in Fig. 1 in comparison with true doses: (**a**) BEAS-2B and (**b**) SVEC4-10EHR1 cells. Each error bar indicates one standard error of the estimated dose. Percent difference of the mean dose estimate from the true dose is written next to the bar. Each mean dose estimate was obtained from three to five independent experiments.

The proportionality of the number of excess γ-H2AX FPC to dose was confirmed. The approximate radiation type-specific proportionality constants for BEAS-2B and SVEC4-10EHR1 cells are shown in Fig. 1 as slopes in units of the number of excess FPC per Gy. After the causative radiation type is identified, the dose can be estimated based on the number of excess γ-H2AX FPC according to the corresponding linear fitting function in Fig. 1. Figure 6 presents the estimated doses from 16 test sample sets for each cell line in comparison with the actual doses. The estimated dose differed by less than 15% from the actual dose.

### γ-H2AX dephosphorylation rate

The repair rate of DSBs implies the severity of cellular damage. Repair becomes difficult when a large number of DNA DSBs are generated locally. Accordingly, high-LET radiations induce slower DNA repair than low-LET radiations. In the report of Ugenskiene et al.^15^, helium-3 particle and X-ray exposures resulted in foci half-lives of approximately 10.7 and 2.9 h, respectively. The residual fraction of initial γ-H2AX foci was around 40% at 24 h after helium-3 particle exposure. Antonelli et al.^8^ found that the half-life of γ-H2AX foci upon X-ray exposure was less than 2 h, and that upon alpha particle exposure was between 4 and 8 h. In addition, approximately 30% of γ-H2AX foci remained after alpha particle exposure. Jezkova et al.^24^ confirmed a half-life between 2 and 4 h from γ-ray exposure and a remaining signal ratio of around 85% even at 4 h after Boron-11 and Neon-20 particle exposures.

In this study, BEAS-2B cells showed the shortest half-lives of 5.22 h when exposed to alpha particles and 0.386 h when exposed to X-rays (*ln 2* $\times$ *B*_*1*_ values in Table 2). Under X-ray exposure, DSB repair proceeded with the dominant (*A*_*2*_ = 0.603) half-life of 4.19 h. SVEC4-10EHR1 cells showed the shortest half-lives of 3.98 h when irradiated by alpha particles and 1.57 h when irradiated by X-rays. Foci remain after 24 h in the portions (*A*_*i*_ in Table 2) that correspond to the long half-lives (*ln 2* $\times$ *B*_*i*_ $\gg$ 24 h in Table 2).

Asaithamby et al.^25^ showed that the repair rate was constant from 10 mGy to 1 Gy of γ-ray doses. Rothkamm and Lobrich^26^ also observed no difference in DNA DSB repair rate at X-ray exposures of 20 mGy to 2 Gy, except for an very slow DNA repair rate at 1.2 mGy. Dose independent repair rate was confirmed also at high doses of 3–90 Gy^27,28^. By contrast, Neumaier et al.^29^ reported a meaningful change in DSB repair rate due to different X-ray doses and mentioned that the repair rate varied depending on the number of DSBs present in a volume. This finding was indicative of the difficult repair of clustered DSBs. In the present study, no significant difference in dephosphorylation rate (or repair rate) was observed after exposure at three doses of 0.2, 0.5 and 1 Gy (Fig. 3). We conclude that DSB repair rate is affected by the DNA damage concentration in a limited volume, and the repair rate remains the same except at exposure to utmost doses.

### Individual dose estimation based on dephosphorylation rate of γ-H2AX

Radiation causes DNA damages by directly transmitting energy to DNA or generating ROS around DNA. These physical or physicochemical processes are hardly interrupted by each other. The repair rate of each focus is also not obstructed by the presence of other foci. Hence, the total number of γ-H2AX foci would be equal to the sum of the numbers generated by individual radiations. In this study, dephosphorylation rate showed dependency on the causative radiation type, which determines the complexity of DNA DSBs. Consistent rates of γ-H2AX dephosphorylation were observed regardless of the number of γ-H2AX foci when exposed to the same type of radiation at different doses up to 1 Gy (Fig. 3). Dephosphorylation of γ-H2AX upon exposure to one type of radiation would proceed at its own rate, regardless of whether the neighbouring foci were formed by the same or different types of radiation. These interpretations are supported by Staaf et al.^6^, who showed via γ-H2AX assay that alpha particles and X-rays affect cells in an additive way. Although some cellular responses following DNA damage were expressed in a different manner from additivity^30^, the total number of foci remained to be the sum of the numbers of γ-H2AX foci formed by individual radiation types.

**Figure 7:**
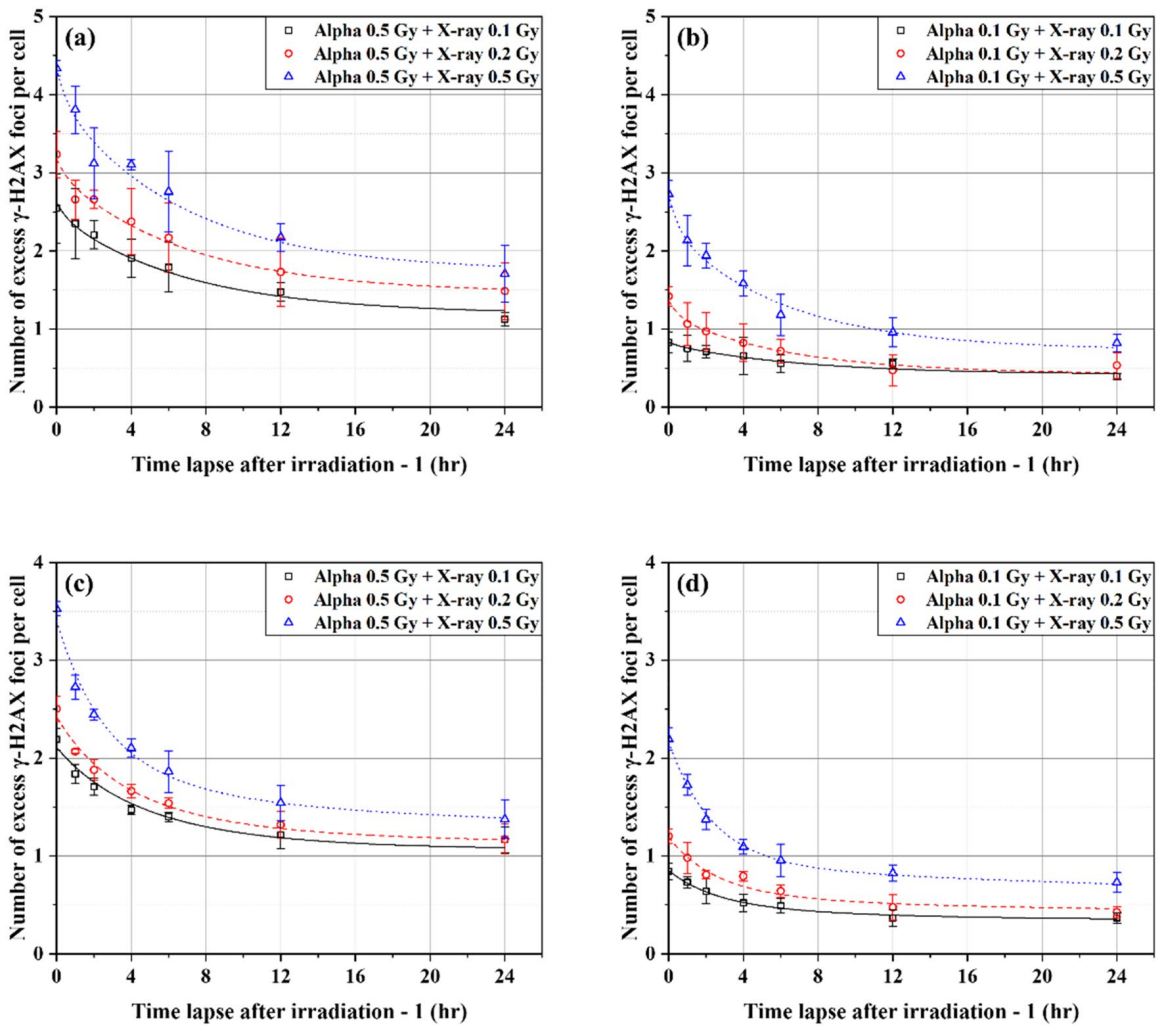
The numbers of excess γ-H2AX foci per cell in BEAS-2B (**a**,**b**) and SVEC4-10EHR1 (**c**,**d**) cells observed over time at 0, 1, 2, 4, 6, 12, and 24 h after mixed beam exposure. Cells were exposed to alpha particles at either 0.5 or 0.1 Gy and then to X-rays at 0.1, 0.2, or 0.5 Gy. Each data point was obtained from four independent experiments.

Figures 7 shows the total numbers of excess γ-H2AX FPC in BEAS-2B (Fig. 7a,b) and SVEC4-10EHR1 (Fig. 7c,d) cells observed at 0, 1, 2, 4, 6, 12, and 24 h after exposure to alpha particles at ${D}_{\alpha }$ (= 0.1, 0.5 Gy) and then X-rays at ${D}_{X}$ (= 0.1, 0.2, 0.5 Gy). Each data point was obtained from four independent experiments of mixed beam exposure. The expected numbers of excess foci attributed to each of alpha particle exposure at ${D}_{\alpha }$ and X-ray exposure at ${D}_{X}$ would decrease over time after irradiation according to Eqs. (1) and (2), respectively, where the values of *A*_*i*_ and *B*_*i*_ depend on the cell line and radiation type, as listed in Table 2.

1 $${\text{Expected}}\;{\text{number}}\;{\text{of}}\;{\text{excess}}\;{\text{foci}}\;{\text{due}}\;{\text{to}}\;{\text{alpha}}\;{\text{particle}}\;{\text{exposure}}\;{\text{at}}\;D_{\alpha } = D_{\alpha } \cdot k_{\alpha } \cdot \left( {A_{1} e^{{ - t/B_{1} }} + A_{2} e^{{ - t/B_{2} }} + A_{3} e^{{ - t/B_{3} }} } \right)_{\alpha }$$

2 $${\text{Expected}}\;{\text{number}}\;{\text{of}}\;{\text{excess}}\;{\text{foci}}\;{\text{due}}\;{\text{to}}\;{\text{X-ray}}\;{\text{exposure}}\;{\text{at}}\;D_{X} = D_{X} \cdot k_{X} \cdot \left( {A_{1} e^{{ - t/B_{1} }} + A_{2} e^{{ - t/B_{2} }} + A_{3} e^{{ - t/B_{3} }} } \right)_{X}$$

Here ${k}_{\alpha }$ and ${k}_{X}$ are the slopes for alpha particles and X-rays, respectively, as shown in Fig. 1.

Assuming that the actual doses of ${D}_{\alpha }$ and ${D}_{X}$ are unknown, the function in Eq. (3) was defined to obtain ${DF}_{\alpha }$ and ${DF}_{X}$ values that best fit the data points in Fig. 7:

3 $$\begin{aligned} &{\text{Total}}\;{\text{number}}\;{\text{of}}\;{\text{excess}}\;{\text{foci}}\;{\text{due}}\;{\text{to}}\;{\text{mixed}}\;{\text{beam}}\;{\text{exposure}} \\ &\quad= {\text{ }}DF_{\alpha } \cdot k_{\alpha } \cdot \left( {A_{1} e^{{ - t/B_{1} }} + A_{2} e^{{ - t/B_{2} }} + A_{3} e^{{ - t/B_{3} }} } \right)_{\alpha } {\text{ }} + {\text{ }}DF_{X} \cdot k_{X} \cdot \left( {A_{1} e^{{ - t//B_{1} }} + A_{2} e^{{ - t/B_{2} }} + A_{3} e^{{ - t/B_{3} }} } \right)_{X} \end{aligned}$$

**Figure 8:**
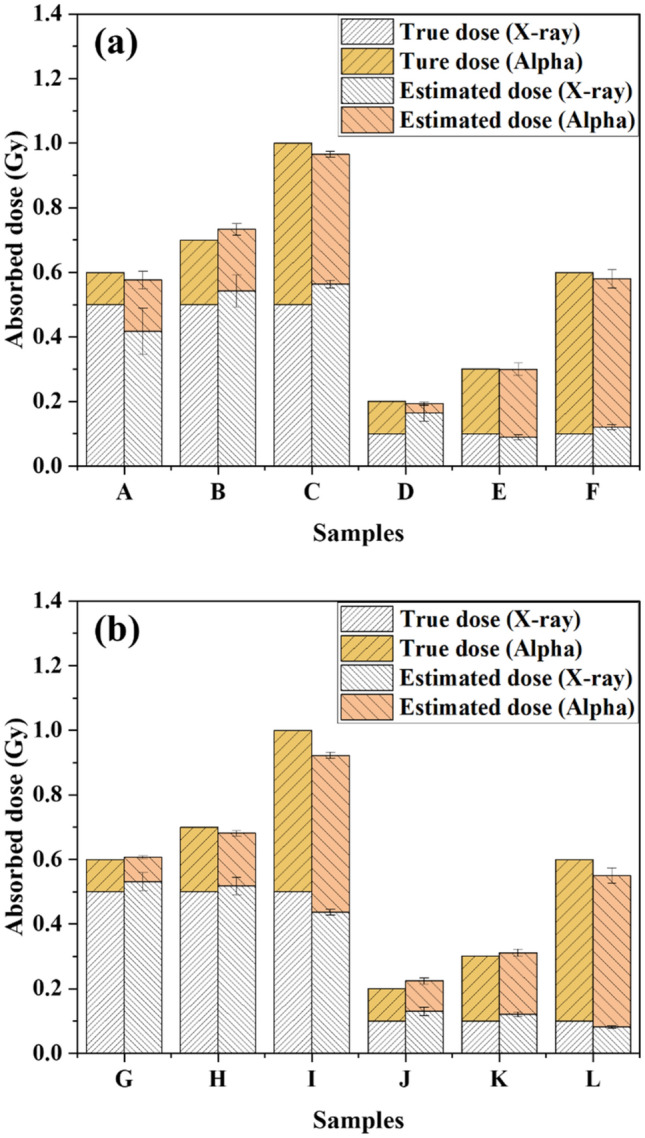
Dose estimates of alpha particles and X-rays that fit the experimental data of excess γ-H2AX foci per cell in (**a**) BEAS-2B and (**b**) SVEC4-10EHR1 cells in Fig. 7 in comparison with the actual doses. Each error bar indicates one standard error of the estimated dose. Each dose estimate was obtained from four or more independent experiments.

Each curve in Fig. 7 fitted the relevant data points with good correlations (R^2^ > 0.95). Figure 8 depicts the comparison between the derived values of ${DF}_{\alpha }$ for alpha particles and ${DF}_{X}$ for X-rays and the corresponding actual doses ${D}_{\alpha }$ and ${D}_{X}$ under six different conditions of mixed beam exposure (0.1 or 0.5 Gy of alpha dose and 0.1, 0.2, or 0.5 Gy of X-ray dose) for each cell line. Individual doses of alpha particles and X-rays were approximated by ${DF}_{\alpha }$ and ${DF}_{X}$, respectively, with less than 12.1% of error, except for the case of (0.1 Gy of alpha dose + 0.1 Gy of X-ray dose) where the individual dose estimates deviated significantly from the actual.

### Application and limitations

Conventional biological dosimetry, which examines the chromosomal abnormalities in blood lymphocytes, is useful in determining systemic exposure levels to penetrating gamma-rays and neutrons. Chromosome abnormalities take 3–7 days to be observed^31^. The γ-H2AX assay has advantage of obtaining analysis results for a large amount of samples in a short time^31^. Compared to other methods, analysis can be performed with less labor and cost, so γ-H2AX assay can be actively used when many people are exposed to radiation. γ-H2AX assay can be utilized to classify the exposed individuals prior to other dosimetric methods. A limitation of the γ-H2AX assay is that the signal is not long-lasting^31,32^. Therefore, the γ-H2AX assay is practical for use as an early triage in a radiological emergency response system. The characteristics of γ-H2AX foci formation in terms of foci size and distribution may find use in mixed-beam exposure incidents where rapid determination of exposure levels via biopsy is required. The application is the theme of future studies.

In this study, the characteristics of γ-H2AX induction were investigated with 2D images of γ-H2AX foci. The cells became thinner than their original thickness (~ 5 μm) during dehydration, and the z-directional resolution was not sufficient to distinguish overlapping foci. The actual geometry of γ-H2AX foci, if obtained from 3D image analysis, could have further informed details of differences in foci size and distribution due to different radiation LETs.

## Conclusion

γ-H2AX assay was adopted to measure DNA DSBs induced by X-ray and alpha particle exposures. The γ-H2AX foci were counted, and their sizes were measured. The average number of excess γ-H2AX foci formed by radiation exposure was highly (R^2^ > 0.95) correlated with the dose. Size distribution was used to identify the causative radiation type. Radiation type (high or low-LET) was identified at more than 80% (27 out of 32 cases) correctness by employing the three criteria based on focus size distribution. The dephosphorylation rate of γ-H2AX was also specific to the radiation type. γ-H2AX dephosphorylation proceeded more slowly upon exposure to alpha particles than to X-rays. Owing to the radiation-type specific dephosphorylation rate of γ-H2AX, doses attributed to individual radiation types could be derived by examining the change rate of total number of γ-H2AX over 24 h after mixed beam exposure. The doses of alpha particles and X-rays were estimated with an error of less than 12.1%, except for the mixed beam at minimum doses.

## Methods

### Cell lines and culture

Experiments were performed with two cell lines, human lung epithelial cells (BEAS-2B) [Catalog No. CRL-9609, American Type Culture Collection (ATCC), Manassas, VA, USA] and mouse vascular endothelial cells (SVEC4-10EHR1) (Catalog No. CRL-2161, ATCC). BEAS-2B cells were cultured in LHC-9 medium (Catalog No. 12680-013, Thermo Fisher Scientific, Waltham, MA, USA) with phenol red indicator (Catalog No. PCS-999-001, ATCC). SVEC4-10EHR1 cells were grown in the mixture of 90% Dulbecco’s Modified Eagle Medium (DMEM) (Catalog No. SH30022.01, Hyclone, UT, USA) and 10% heat-inactivated fetal bovine serum (Catalog No. 30-2020, ATCC). BEAS-2B and SVEC4-10EHR1 cells in flasks were incubated at 37 °C with 5% humidity and 10% CO_2_. Culture medium was changed at least three times a week. The cells were seeded on a 4 μm-thick Mylar-bottomed dish 1 day prior to irradiation.

### Irradiation

Cells were exposed to alpha particles and X-rays individually or were sequentially exposed in the order of alpha particles and X-rays. Alpha particle irradiation was conducted by using the SNU-ALPHACELL, the alpha particle irradiation system at Seoul National University (SNU) made for studying cellular response to alpha emissions from natural sources^21^. Cells were exposed to alpha particles emitted from an Americium-241 disc source at a distance of 30 mm. X-ray exposure was made in the SNU-HARDX facility^33^, where the YXLON 450-D08 beam tube (Germany) operated at 150 kVp and 3.60 mA. Both alpha particle and X-ray exposures were made at a constant rate of 0.356 Gy/min to deliver doses up to 1 Gy. One of the authors performed a blind test to identify the radiation type and dose with the irradiated samples.

### γ-H2AX assay

Cells were detached from Mylar dishes 0 to 24 h after irradiation. Cells harvested from Mylar dishes were centrifuged to be attached onto a slide glass in the cyto-centrifuge (Catalog No. Rotofix 32A, Hettich, Tuttlingen, Germany). Hydrophobic barrier lines were drawn on the slide glass using a PAP pen to prevent reagent loss during centrifuge operation. Samples were handled carefully to avoid cell loss during the entire process. The cyto-centrifugation process took 1 h. The detailed subsequent experimental process was described in Lee et al.^34^. The attached cells were washed with PBST, the Dulbecco’s phosphate buffered saline (DPBS) (Catalog No. 14040-117, Gibco, Grand Island, NY, USA) containing 0.05% tween 20 (Catalog No. T9100-010, GenDEPOT), and fixed in 4% paraformaldehyde (Catalog No. 163-20145, Wako Pure Chemical Industries, Ltd., Japan). After fixation, the cells were washed twice with ice-cold PBST, permeabilized with 1% Triton X-100 (Catalog No. T9500-010, GenDEPOT, TX, USA) solution for 10 min, washed again three times with PBST, and blocked with 10% bovine serum albumin (BSA) (Catalog No. A0100-010, GenDEPOT) in PBST. These samples were stained with anti-γ-H2AX phosphor S139 antibody (Catalog No. Ab2893, Abcam, Cambridge, UK) and 1% BSA in PBST solution. After 1 h, the cells were washed with PBST three times and treated with goat anti-rabbit IgG H&L fluorescein isothiocyanate antibody (Catalog No. Ab6717, Abcam) in 1% BSA solution for 1 h. After immunostaining, the cells were washed three times with PBST, and stained with 4′6-diamidino-2-phenylindole in a fluoroshield mounting medium (DAPI) (Catalog No. Ab104139, Abcam). A cover slip was put over the cells with care not to let air bubbles in. At least three independent experiments were conducted for one data value, and about 500 to 700 cells per individual sample were analyzed.

### Foci counting and size measurement

Images of γ-H2AX foci were taken using a fluorescence microscope (Catalog No. BX53F, Olympus, Tokyo, Japan) with a 40 × UplanSApo objective. The number of γ-H2AX foci per cell (FPC) was counted by employing the open-source software “CellProfiler” (version 2.1.1, Broad Institute’s Imaging Platform, USA). The number of excess FPC in treated cells was obtained by subtracting the number of FPC in control cells from the count in treated cells. Dephosphorylation of γ-H2AX was examined by counting the foci at different elapsed time points of 0, 1, 2, 4, 6, 12, and 24 h after radiation exposure.

The CellProfiler formed images with a pixel size of 0.25595 μm. The area of each focal image in the cells was measured and classified into nine groups, ranging from 0.205 μm^2^ to 16.7 μm^2^. Foci in control cells were also sorted by size. For each size group, the number of foci in control cells was subtracted from the number of foci in treated cells to obtain the number of excess foci in the treated cells. A normalized size distribution was determined with the numbers of excess foci sorted by size for each radiation treatment condition (radiation type and dose) (Supplementary Fig. 1).

### Statistical analysis

Two-tailed student’s t-test was performed to determine whether the difference between observed values is significant to recognize the influence of treatment and its condition. The significance was judged at 95% confidence level of null hypothesis (p < 0.05).

## Supplementary Information

Supplementary Figure 1.
