# Supplementary material for: Characterization of γ-H2AX foci formation under alpha particle and X-ray exposures for dose estimation
Source: Sci Rep. 2022 Mar 8;12:3761. doi: 10.1038/s41598-022-07653-y (PMC8904799; doi:10.1038/s41598-022-07653-y)
Supplement: Supplementary file 1 — Supplementary Figure 1. [file 41598_2022_7653_MOESM1_ESM.docx]

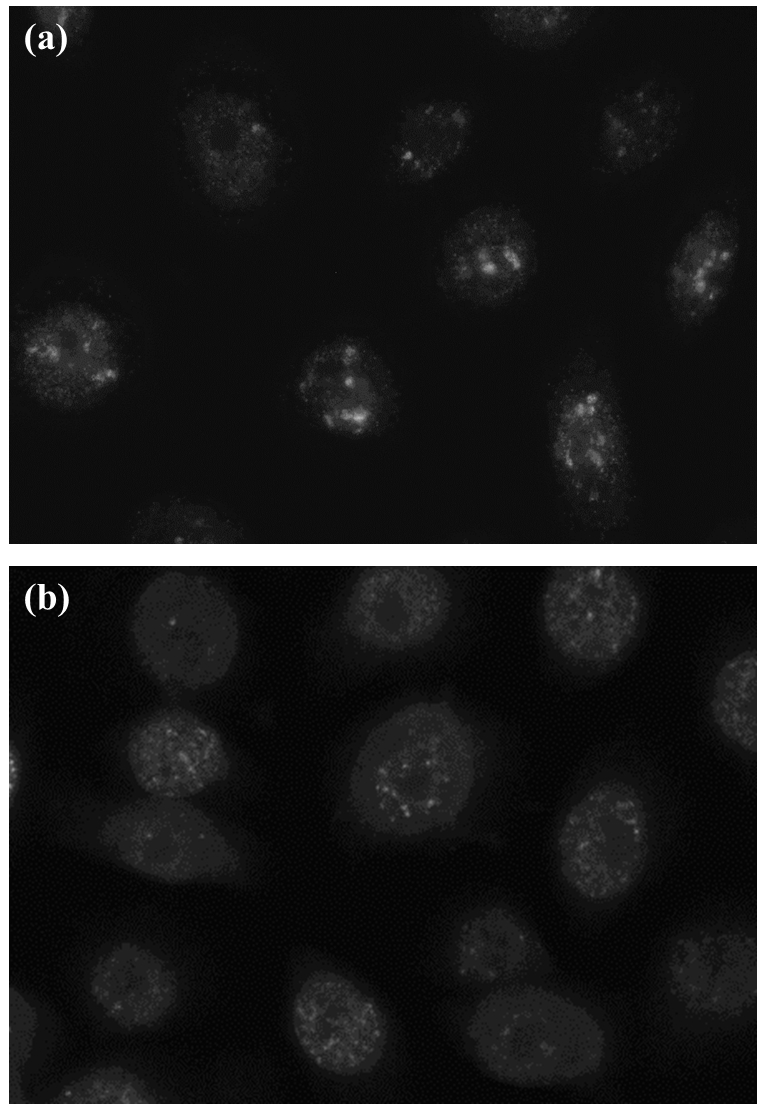


**Supplementary Figure 1.** Actual γ-H2AX foci images in BEAS-2B cells induced by (a) alpha particles and (b) X-rays. Alpha particle made larger and brighter foci.
